# Diagnostic value of expired gas analysis in heart failure with preserved ejection fraction

**DOI:** 10.1038/s41598-023-31381-6

**Published:** 2023-03-16

**Authors:** Yuki Saito, Masaru Obokata, Tomonari Harada, Kazuki Kagami, Makoto Murata, Hidemi Sorimachi, Toshimitsu Kato, Naoki Wada, Yasuo Okumura, Hideki Ishii

**Affiliations:** 1 Department of Cardiovascular Medicine, Gunma University Graduate School of Medicine, 3-39-22 Showa-Machi, Maebashi, Gunma 371-8511 Japan; 2 Division of Cardiology, Department of Medicine, Nihon University School of Medicine, Tokyo, Japan; 3 Division of Cardiovascular Medicine, National Defense Medical College, Tokorozawa, Saitama Japan; 4 Department of Cardiology, Gunma Prefectural Cardiovascular Center, Maebashi, Gunma Japan; 5 Department of Rehabilitation Medicine, Gunma University Graduate School of Medicine, Maebashi, Gunma Japan

**Keywords:** Cardiology, Diagnosis

## Abstract

Cardiopulmonary exercise testing (CPET) may potentially differentiate heart failure (HF) with preserved ejection fraction (HFpEF) from noncardiac causes of dyspnea (NCD). While contemporary guidelines for HF recommend using CPET for identifying causes of unexplained dyspnea, data supporting this practice are limited. This study aimed to determine the diagnostic value of expired gas analysis to distinguish HFpEF from NCD. Exercise stress echocardiography with simultaneous expired gas analysis was performed in patients with HFpEF (n = 116) and those with NCD (n = 112). Participants without dyspnea symptoms were also enrolled as controls (n = 26). Exercise capacity was impaired in patients with HFpEF than in controls and those with NCD, evidenced by lower oxygen consumption (VO_2_), but there was a substantial overlap between HFpEF and NCD. Receiver operating characteristic curve analyses showed modest diagnostic abilities of expired gas analysis data in differentiating individuals with HFpEF from the controls; however, none of these variables clearly differentiated between HFpEF and NCD (all areas under the curve < 0.61). Expired gas analysis provided objective assessments of exercise capacity; however, its diagnostic value in identifying HFpEF among patients with symptoms of exertional dyspnea was modest.

## Introduction

Heart failure with preserved ejection fraction (HFpEF) accounts for more than half of patients with heart failure (HF), and its prevalence is expected to increase in tandem with the aging of the general population and increasing burden of cardiac and metabolic comorbidities^1,2^. The diagnosis of HFpEF is straightforward when patients demonstrate apparent signs of congestion, such as peripheral edema, jugular venous distention, elevated natriuretic peptide levels, or pulmonary congestion on chest radiography^3,4^. However, the diagnosis is challenging in patients presenting with chronic dyspnea with no or a lesser degree of congestion because the left ventricular (LV) filling pressure, which is the primary abnormality of HF, is often normal in these patients when assessed at rest. However, the LV filling pressure dramatically increases only during physiological stress, such as during exercise^5–8^. Accumulating evidence has demonstrated the use of exercise stress testing (invasive hemodynamic exercise test or exercise stress echocardiography) for identifying abnormalities that develop during exercise^5,6,9,10^. Exercise stress testing is now recommended for the diagnostic evaluation of HFpEF^11–13^.

Exercise intolerance is the primary manifestation of HFpEF^7,14,15^. Cardiopulmonary exercise testing (CPET) provides an objective assessment of exercise capacity by measuring peak oxygen consumption (VO_2_)^16–18^. CPET is also valuable for evaluating the integrity of exercise physiology involving the pulmonary, cardiovascular, and muscular systems and may potentially differentiate HFpEF from noncardiac causes of dyspnea (NCD)^12^. Contemporary guidelines for HF recommend the use of CPET for identifying causes of unexplained dyspnea and there is an increasing interest in exercise stress echocardiography combined with expired gas analysis (CPET imaging)^18^. However, data regarding the diagnostic value of expired gas analysis are limited^19–21^.

Accordingly, we performed comprehensive exercise stress echocardiography with simultaneous expired gas analysis in patients with unexplained dyspnea to explore this. We also enrolled patients without exertional dyspnea or HF to investigate the effects of the presence or absence of shortness of breath on the diagnostic ability of expired gas analysis.

## Methods

### Study population

Consecutive subjects referred to the echocardiographic laboratory in Gunma University Hospital for exercise stress echocardiography for the evaluation of exertional dyspnea between November 2019 and March 2022 were retrospectively identified. The diagnosis of HFpEF was defined using the Heart Failure Association Pre-test assessment, Echocardiography and natriuretic peptide, Functional testing, Final etiology (HFA-PEFF) algorithm in Steps 1–3^12^. In brief, the HFA-PEFF score was calculated as the sum of echocardiographic functional (age-specific cut-offs for early diastolic mitral annular velocity [e′] velocity, early transmitral flow velocity [E]/e′ ratio, tricuspid regurgitation [TR] velocity, and longitudinal strain: maximum 2 points), morphological (rhythm-specific left atrial [LA] volume, relative wall thickness, and sex-specific measures of LV mass: maximum 2 points), and natriuretic peptide (maximum 2 points) domains. Subsequently, two or three points were added depending on the E/e′ ratio and TR velocity during exercise stress echocardiography. The diagnosis of HFpEF was confirmed if the combined score from Steps 2 and 3 was ≥ 5 points. Patients who did not meet the HFA-PEFF criteria were categorized as having noncardiac dyspnea. Patients with an ejection fraction (EF) of < 50%; significant left-sided valvular heart disease (> moderate regurgitation, > mild stenosis); infiltrative, restrictive, or hypertrophic cardiomyopathy; and non-group II pulmonary arterial hypertension or exercise-induced pulmonary hypertension without elevation in E/e′ (pulmonary arterial mean pressure [mPAP] of > 30 mmHg during exercise with a total pulmonary resistance [i.e., mPAP/cardiac output {CO}] of > 3 mmHg･min/L) were excluded^22^. We also included 26 participants with no dyspnea in daily activities who underwent exercise echocardiography for the evaluation of exercise capacity and cardiac reserve as a comparator group (controls).

### Ethical declarations

The study was approved by our Institutional Review Board with the waiver of informed consent because its retrospective design (HS2022-110, Gunma University Hospital, Clinical Research Review Board), and was performed in accordance with the Declaration of Helsinki and the ethical guidelines for medical and biological research involving human subjects in Japan. Participants were guaranteed the opportunity to refuse the study using an opt-out approach (details can be found on the website; https://ciru.dept.showa.gunma-u.ac.jp/guidance/storage-sample/pdf/2022-110.pdf). All authors have read and agree to the manuscript as written.

### Assessment of ventricular structure and function

Transthoracic echocardiography was performed by experienced sonographers using a commercially available ultrasound system (Vivid E95; GE Healthcare, Horten, Norway). LV systolic function at rest and during exercise was assessed based on the EF and systolic mitral annular tissue velocity at the septal annulus (mitral s′). LV diastolic function was assessed using E, e′, and septal E/e′ ratio. Stroke volume was determined from the LV outflow dimension and pulse Doppler profile, and CO was calculated from the product of the heart rate and stroke volume. Right ventricular (RV) systolic function was assessed using the systolic tissue velocity at the lateral tricuspid annulus (TV s′). Right atrial pressure (RAP) was estimated from the diameter of the inferior vena cava and its respiratory changes. The pulmonary artery systolic pressure (PASP) was calculated as 4 × (peak TR velocity)^2^ + estimated RAP. All Doppler measurements represented a mean of ≥ 3 beats.

### Exercise stress echocardiography with simultaneous expired gas analysis

All participants underwent supine cycle ergometry echocardiography, starting at 20 W for 5 min, with increments of 20 W in 3-min stages to participant-reported exhaustion. Echocardiographic images were obtained at baseline and during all stages of exercise. Expired gas analysis was performed simultaneously with echocardiography at rest and throughout exercise in all participants. Breath-by-breath VO_2_, carbon dioxide production (VCO_2_), tidal volume (V_T_), respiratory rate, and minute ventilation (V_E_ = V_T_ × respiratory rate) were measured continuously as previously described (AE-100i, MINATO Medical Science, Osaka, Japan)^7,15^. Percent predicted peak VO_2_ was estimated using the Wasserman-Hansen equation. The objective effort was estimated by the respiratory exchange ratio (RER = VCO_2_/VO_2_), and the ventilatory efficiency was assessed by the slope of V_E_ to VCO_2_ (V_E_ vs. VCO_2_ slope). All analyses of ventilation and gas exchange data were performed offline in a blinded fashion by one investigator (KK).

### Statistical analysis

Data are reported as mean (standard deviation), median (interquartile range), or number (%) unless otherwise specified. Between-group differences were compared using one-way analysis of variance, the Kruskal–Wallis test, or chi-square test, as appropriate. Tukey’s honestly significant difference test or the Steel–Dwass test was used to adjust for multiple testing. The diagnostic ability was determined using receiver operating characteristic curves. All tests were two-sided, with statistical significance set at P < 0.05. All statistical analyses were performed using JMP 13.0.0 (SAS Institute, Cary, NC, USA).

## Results

### Baseline clinical characteristics

Of the 228 participants with exertional dyspnea, 116 met the criteria for HFpEF, and 112 patients were classified as having NCD. Patients with HFpEF were older than those in the other groups; however, the sex was similar among the groups (Table 1). Compared with patients with NCD, patients with HFpEF had a greater body mass index and a higher prevalence of coronary artery disease, diabetes mellitus, systemic hypertension, and atrial fibrillation and were treated with neurohormonal blockers and diuretics more frequently. Of the 112 patients with NCD, 41 (37%) had chronic obstructive lung disease or interstitial lung disease. As expected, B-type natriuretic peptide levels were the highest and red blood cell counts and levels of hemoglobin and hematocrit were the lowest in patients with HFpEF. Estimated glomerular filtration rate was lower in HFpEF than in NCD. Patients with HFpEF had a larger LV mass index, LA volume, and E/e′ ratio than those in the other groups, consistent with LV diastolic dysfunction (Table 1). The LV diastolic dimension, LVEF, and RAP were similar across groups. Regarding expired gas data at rest, VO_2_, respiratory rate, V_E_, and V_T_ were similar across groups.

**Table 1:** Baseline characteristics.

	Controls (n = 26)	NCD (n = 112)	HFpEF (n = 116)	P value
Age (years)	68 ± 6	65 ± 13	74 ± 7*^†^	< 0.0001
Female, n (%)	13 (50)	69 (62)	67 (58)	0.53
Body mass index (kg/m^2^)	24.1 ± 4.6	23.6 ± 6.0	24.8 ± 6.4^†^	0.04
Comorbidities				
Coronary disease, n (%)	2 (8)	4 (4)	16 (14)^†^	0.02
Diabetes mellitus, n (%)	4 (15)	16 (14)	35 (30)^†^	0.01
Hypertension, n (%)	17 (65)	66 (59)	96 (83)^†^	0.0003
Atrial fibrillation, n (%)	6 (23)	4 (4)*	26 (23)^†^	< 0.0001
Medications				
ACEI or ARB, n (%)	11 (42)	32 (29)	54 (47)^†^	0.02
Beta-blocker, n (%)	7 (27)	12 (11)*	37 (32)^†^	0.0003
Loop diuretics, n (%)	2 (8)	10 (9)	41 (36)*^†^	< 0.0001
Laboratories				
BNP (pg/mL), n = 144	48 (25, 71)	32 (16, 58)	112 (46, 213)*^†^	< 0.0001
NT-pro BNP (pg/mL), n = 84	159 (105, 383)	128 (68, 182)	511 (251, 1566)^†^	< 0.0001
Red blood cell count, ×10^6^/μL	4.48 ± 0.57	4.34 ± 0.52	4.09 ± 0.61*^†^	0.001
Hemoglobin, g/dL	13.6 ± 1.8	13.1 ± 1.5	12.3 ± 1.8*^†^	0.0006
Hematocrit, (%)	41.7 ± 5.3	40.4 ± 4.3	37.9 ± 5.5*^†^	0.0003
eGFR, mL/min/1.73 m^2^	66.0 ± 18.8	67.7 ± 17.7	56.7 ± 22.7^†^	0.0008
Vital signs				
Heart rate (bpm)	71 ± 15	75 ± 13	73 ± 13	0.37
Systolic BP (mmHg)	130 ± 34	129 ± 20	127 ± 20	0.84
Saturation (%)	97 ± 1	97 ± 2	97 ± 2	0.20
LV structure and function				
LV diastolic dimension (mm)	44 ± 5	43 ± 6	45 ± 6	0.08
LV mass index (g/m^2^)	81 ± 14	76 ± 20	93 ± 23*^†^	< 0.0001
LV ejection fraction (%)	64 ± 4	64 ± 6	64 ± 7	0.83
LA volume index (mL/m^2^)	30 (24, 38)	23 (19, 29)*	36 (30, 47)*^†^	< 0.0001
E/e′ ratio (septal)	9.7 ± 2.8	9.4 ± 2.6	14.4 ± 5.3*^†^	< 0.0001
PASP (mmHg)	22 ± 7	22 ± 6	25 ± 10	0.26
RAP (mmHg)	4 ± 2	4 ± 2	4 ± 3	0.14
Expired gas data				
VO_2_ (mL/kg/min)	4.1 ± 1.0	4.1 ± 1.4	3.8 ± 0.9	0.13
Respiratory rate (/min)	15.5 ± 5.3	16.8 ± 5.6	16.4 ± 5.9	0.50
V_E_ (L/min)	9.6 ± 2.8	9.9 ± 3.7	9.3 ± 2.5	0.76
V_T_ (mL)	659 ± 230	597 ± 188	613 ± 205	0.45

Data are mean ± SD, median (interquartile range), or n (%). Final column reflects overall group differences. *p < 0.05 vs. Controls; ^†^p < 0.05 vs. NCD.

ACEI, angiotensin-converting enzyme inhibitors; ARB, angiotensin-receptor blockers; BNP, B-type natriuretic peptide; BP, blood pressure; E/e′ ratio, the ratio of early diastolic mitral inflow to mitral annular tissue velocities; eGFR, estimated glomerular filtration rate; HFpEF, heart failure with preserved ejection fraction; LA, left atrial; LV, left ventricular; NCD, non-cardiac dyspnea; NT-proBNP, N-terminal pro B-type natriuretic peptide; PASP, pulmonary artery systolic pressure; and RAP, right atrial pressure; VE_,_ minute ventilation; VO_2_, oxygen consumption; V_T,_ tidal volume.

### Echocardiographic measures during submaximal exercise

**Figure 1:**
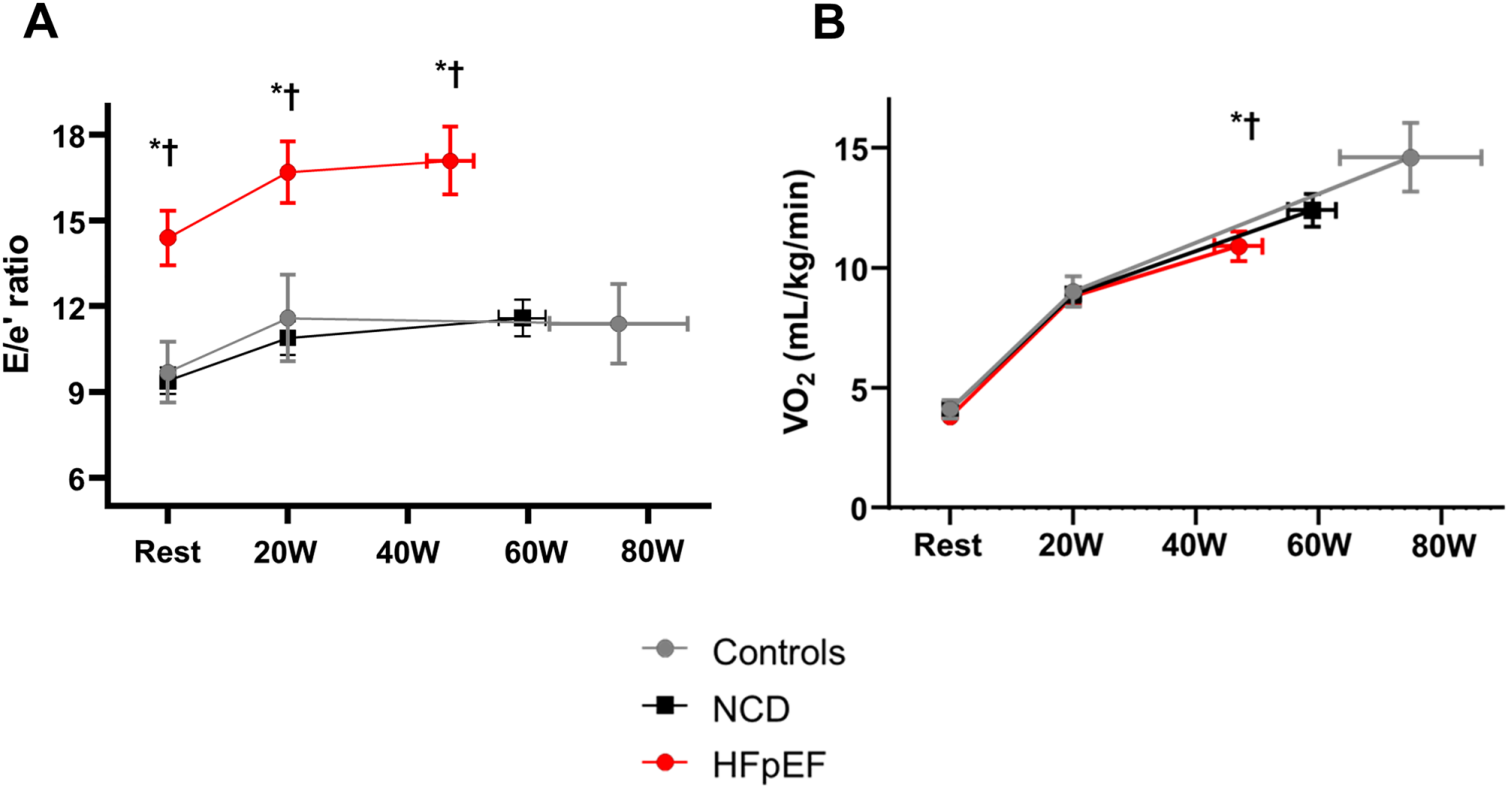
(**A**,**B**) Changes in the early transmitral flow velocity/early diastolic mitral annular velocity ratio and peak oxygen consumption at rest and during low-level (20 W) and peak exercise in patients with HFpEF and NCD and the controls. *P < 0.05 vs. Controls; ^†^P < 0.05 vs. NCD. HFpEF, heart failure with preserved ejection fraction; NCD, noncardiac dyspnea.

During the matched submaximal (20 W) exercise, the heart rate, systolic blood pressure, oxygen saturation, and LVEF were similar across the groups (Table 2). Compared with other groups, patients with HFpEF had a significantly higher E-wave, lower mitral e′ velocity, and higher E/e′ ratio during submaximal exercise (Fig. 1A), indicative of worsening LV diastolic function. Biventricular systolic function (mitral s′ and TV s′) was lower, and PASP was higher in patients with HFpEF than in those with NCD.

**Table 2:** Echocardiographic measures during matched 20W exercise.

	Controls (n = 26)	NCD (n = 112)	HFpEF (n = 116)	P value
Vital signs				
Heart rate (bpm)	93 ± 18	95 ± 15	93 ± 17	0.34
Systolic BP (mmHg)	143 ± 31	152 ± 28	147 ± 26	0.22
Saturation (%)	96 ± 2	95 ± 4	95 ± 3	0.35
Echocardiographic measures				
LV ejection fraction (%)	64 ± 4	64 ± 6	64 ± 7	0.83
E-wave (cm/s)	72 ± 14	63 ± 18	80 ± 27*^†^	< 0.0001
Septal mitral e′ (cm/s)	7.9 ± 2.0	6.9 ± 1.8*	5.8 ± 1.7*^†^	< 0.0001
Septal mitral s′ (cm/s)	7.7 ± 1.6	7.6 ± 1.7	6.4 ± 1.6*^†^	< 0.0001
E/e′ ratio (septal)	11.6 ± 3.7	10.9 ± 3.1	16.7 ± 5.9*^†^	< 0.0001
Cardiac output (L/min)	5.5 ± 1.4	5.7 ± 1.7	5.7 ± 1.6	0.88
TV s′ (cm/s)	13.2 ± 2.9	13.6 ± 2.5	12.1 ± 3.0^†^	0.0005
PASP (mmHg)	38 ± 12	35 ± 11	40 ± 12^†^	0.001

Data are mean ± SD or median (interquartile range). Final column reflects overall group differences. *p < 0.05 vs. Controls; ^†^p < 0.05 vs. NCD.

TV, tricuspid valvular; and other abbreviations as in Table 1.

### Echocardiographic measures and expired gas data during peak exercise

**Figure 2:**
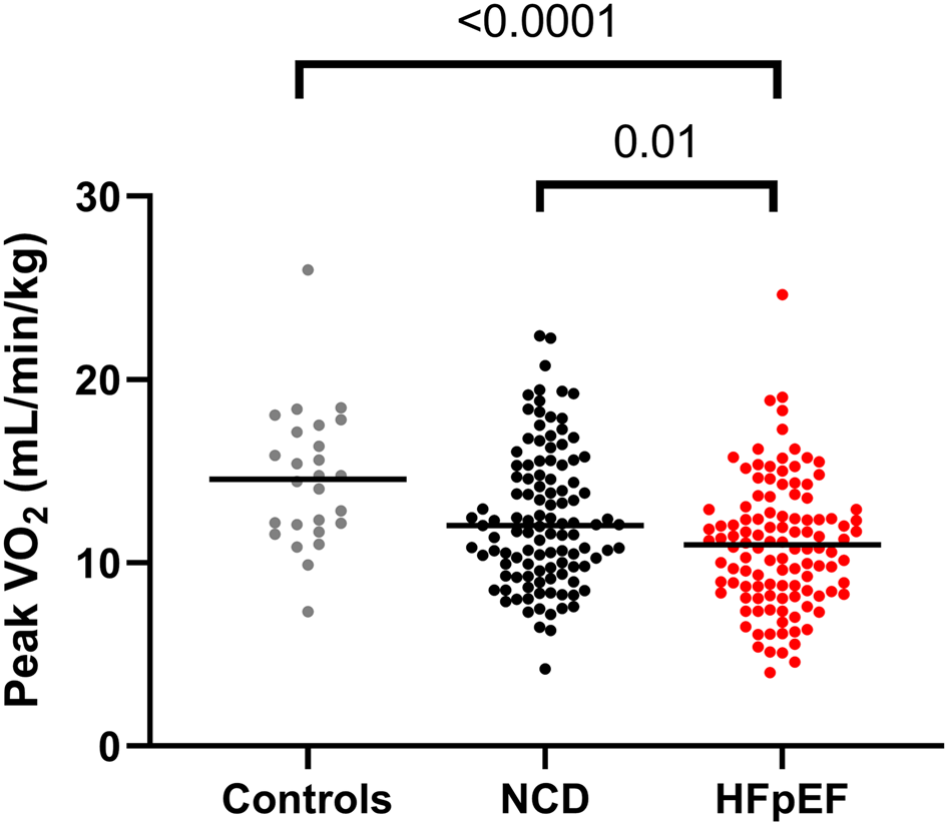
Comparisons in peak oxygen consumption among patients with HFpEF, those with NCD, and the controls. Abbreviations as in Fig. 1.

Exercise capacity was impaired in patients with HFpEF compared with the other groups, as evidenced by lower peak exercise workload, shorter exercise duration, and lower peak VO_2_ (Table 3, Fig. 1B). Patients with NCD had worse exercise capacity than the controls. Although the peak VO_2_ was lower in the HFpEF group than in the NCD group, there was a substantial overlap between the groups (Fig. 2). During peak exercise, systolic blood pressure and oxygen saturation were similar across the groups; however, the heart rate was significantly lower in patients with HFpEF than in the controls. Compared with the other groups, patients with HFpEF had a lower mitral e′ and higher E/e′ ratio during peak exercise (Fig. 1A). LV and RV systolic functions (mitral s′ and TV s′) were the poorest in patients with HFpEF. Regarding expired gas data during peak exercise, the NCD and HFpEF groups demonstrated a lower V_T_ and worse ventilatory efficiency (higher V_E_ vs. VCO_2_ slope and minimum V_E_/VCO_2_) than the control group, and O_2_ pulse and RER were lower in the HFpEF group than in the controls. However, there was no difference in these expired gas parameters between the NCD and HFpEF groups except indexed VO_2_.

**Table 3:** Echocardiographic measures and expired gas data during peak exercise.

	Controls (n = 26)	NCD (n = 112)	HFpEF (n = 116)	P value
Peak Watts (W)	75 ± 31	59 ± 21*	47 ± 22*^†^	< 0.0001
Exercise time (min)	11.8 ± 4.2	9.4 ± 3.0*	7.7 ± 3.3*^†^	< 0.0001
Vital signs				
Heart rate (bpm)	121 ± 23	116 ± 22	110 ± 24*	0.02
Systolic BP (mmHg)	174 ± 34	166 ± 30	161 ± 33	0.11
Saturation (%)	95 ± 3	94 ± 5	94 ± 4	0.53
Echocardiographic measures				
LV ejection fraction (%)	71 ± 5	71 ± 7	69 ± 8	0.21
E-wave (cm/sec)	122 ± 21	111 ± 24	127 ± 30^†^	0.0002
Septal mitral e′ (cm/s)	11.2 ± 2.9	9.9 ± 2.4	7.9 ± 2.1*^†^	< 0.0001
Septal mitral s′ (cm/s)	8.5 ± 1.7	9.0 ± 2.1	7.3 ± 1.8*^†^	< 0.0001
E/e′ ratio (septal)	11.4 ± 3.2	11.6 ± 3.0	17.1 ± 6.1*^†^	< 0.0001
Cardiac output (L/min)	7.4 ± 1.4	7.0 ± 2.0	6.6 ± 2.0	0.10
TV s′ (cm/sec)	14.7 ± 2.6	14.8 ± 3.0	12.7 ± 3.3*^†^	< 0.0001
PASP (mmHg)	46 ± 12	42 ± 11	44 ± 12	0.26
Expired gas data				
VO_2_ (mL/min/kg)	14.6 ± 3.7	12.4 ± 3.7*	10.9 ± 3.4*^†^	< 0.0001
O_2_ pulse (mL/min/beat)	7.6 ± 3.0	6.5 ± 2.3	6.0 ± 2.0*	0.04
RER	1.19 ± 0.16	1.12 ± 0.15	1.10 ± 0.13*	0.02
Respiratory rate (/min)	30 ± 6	32 ± 8	33 ± 8	0.36
V_E_ (L/min)	37.3 ± 14.1	31.7 ± 9.9	29.9 ± 9.8*	0.03
V_T_ (mL)	1250 ± 456	991 ± 314*	935 ± 322*	0.003
Minimum V_E_/VCO_2_ (ratio)	33.5 ± 6.5	39.5 ± 9.6*	41.0 ± 8.8*	< 0.0001
V_E_ vs. VCO_2_ slope	31.4 ± 7.6	37.0 ± 9.5*	39.5 ± 10.1*	< 0.0001

Data are mean ± SD or median (interquartile range). Final column reflects overall group differences. *p < 0.05 vs. Controls; ^†^p < 0.05 vs. NCD.

RER, respiratory exchange ratio; VCO_2_, carbon dioxide volume; and other abbreviations as in Tables 1 and 2.

### Diagnostic ability of expired gas parameters during peak exercise to identify HFpEF

**Figure 3:**
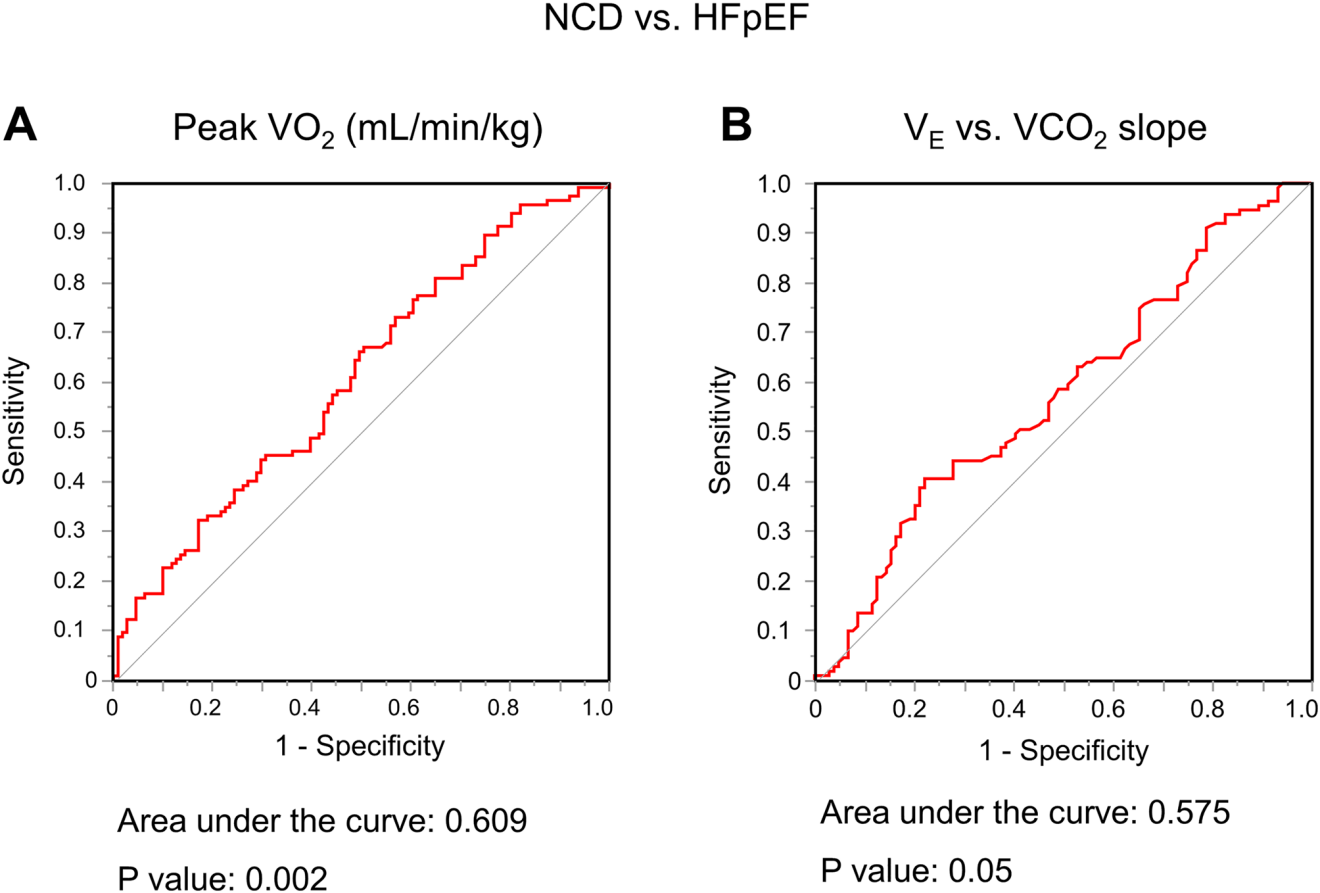
Receiver-operating characteristic curves of peak oxygen consumption (VO_2_) (**A**) and minute ventilation (V_E_) vs. carbon dioxide production (VCO_2_) slope (**B**) for distinguishing HFpEF from NCD. Abbreviations as in Fig. 1.

Receiver operating characteristic curve analyses showed high discriminatory abilities of expired gas parameters during peak exercise in distinguishing individuals with HFpEF from the controls (Table 4). The V_E_/VCO_2_ slope demonstrated highest diagnostic power (area under the curve [AUC] 0.801, P < 0.0001), followed by the indexed and absolute VO_2_. However, the diagnostic abilities of the expired gas parameters in discriminating HFpEF and NCD were limited (all AUCs < 0.61, Fig. 3). Even excluding NCD with chronic obstructive pulmonary disease or interstitial lung disease (n = 41), the diagnostic abilites of expired gas data in distinguishing HFpEF from NCD were modest.

**Table 4:** Diagnostic accuracy of expired gas data.

	Controls vs. HFpEF AUC	P value	NCD vs. HFpEF AUC	P value	NCD without ILD or COPD vs. HFpEF AUC	P value
Peak data						
Saturation (%)	0.540	0.38	0.527	0.41	0.553	0.09
VO_2_ (mL/min)	0.734	< 0.0001	0.601	0.009	0.598	0.02
VO_2_ (mL/min/kg)	0.769	< 0.0001	0.609	0.002	0.623	0.002
%Predicted VO_2_ (%)	0.668	0.007	0.496	0.89	0.544	0.21
O_2_ pulse (mL/min/beat)	0.655	0.001	0.546	0.11	0.542	0.19
VCO_2_ (mL/min)	0.745	< 0.0001	0.596	0.01	0.590	0.02
Respiratory ratio (/min)	0.581	0.09	0.532	0.38	0.457	0.27
V_E_ (L/min)	0.659	0.003	0.555	0.15	0.511	0.72
V_T_ (mL)	0.708	0.0001	0.566	0.18	0.539	0.52
V_E_ vs. VCO_2_ slope	0.801	< 0.0001	0.575	0.05	0.625	0.001
Minimum V_E_/VCO_2_ (ratio)	0.799	< 0.0001	0.567	0.24	0.628	0.005
V_D_/V_T_ (ratio)	0.679	0.003	0.536	0.32	0.594	0.009

ILD, interstitial lung disease; COPD, chronic obstructive pulmonary disease; and other abbreviations as in Tables 1 and 2.

### Sensitivity analyses

Sensitive analysis excluding patients with AF demonstrated essentially similar results to those obtained in the primary analysis (Supplemental Tables 1–3). When excluding patients younger than 70 years from control and NCD groups for age-matched comparisons, we found similar results to the primary analysis with modest diagnostic abilities of the expired gas parameters in discriminating HFpEF from NCD (Supplemental Tables 4–6). We also performed a sensitivity analysis excluding patients with elevated natriuretic peptide levels (B-type natriuretic peptide > 35 pg/mL or N-terminal pro B-type natriuretic peptide > 125 pg/mL) from control subjects and found slightly better diagnostic abilities of expired gas parameters in differentiating HFpEF from controls (Supplemental Tables 7–9).

## Discussion

In the present study, we examined the diagnostic ability of expired gas data to identify HFpEF in patients with unexplained dyspnea. We demonstrated that echocardiography-based LV diastolic reserve was impaired in patients with HFpEF compared with those having NCD and the controls. Expired gas parameters during exercise were worse in patients with HFpEF and NCD than in the controls. Although the peak VO_2_ and V_E_/VCO_2_ slope showed high diagnostic accuracy in differentiating HFpEF from the controls, these parameters were less robust in distinguishing HFpEF from NCD. The current data suggest the limited diagnostic value of expired gas data for identifying HFpEF among dyspneic patients.

Exercise intolerance is a major manifestation of HFpEF^7,23^. HFpEF is a syndrome characterized by multiple reserve limitations, and abnormalities in cardiac, pulmonary, vascular, and peripheral reserves can contribute to reduced exercise capacity^7,15,23–25^. CPET provides valuable insights into the integrity of exercise physiology involving the pulmonary, cardiovascular, and muscular systems and may have the potential to differentiate HFpEF from NCD. There are limited data on the diagnostic value of CPET for HFpEF^19–21^. Reddy et al. performed CPET in invasively proven HFpEF with NCD as a comparator^19^. The authors revealed that multiple CPET variables were predictive of the presence of HFpEF, including abnormal heart rate recovery, reduced VO_2_, and low O_2_ pulse; however, none of these variables accurately discriminated HFpEF from noncardiac dyspnea^19^. Nedeljkovic et al. examined the diagnostic value of CPET for HFpEF in 87 patients with hypertension and exertional dyspnea and found that a higher V_E_ vs. VCO_2_ slope provided an almost perfect diagnostic ability (AUC: 0.99, cut-off: 32.95, sensitivity: 100%, and specificity: 91%)^21^. The small number of cases (n = 8, 9.2%) might have biased the overall results, and the exclusion of chronic obstructive pulmonary disease might have overestimated the diagnostic value of the V_E_ vs. VCO_2_ slope.

In the current study, we performed expired gas analysis simultaneously with exercise stress echocardiography in patients with HFpEF and compared it with that of individuals with NCD and the controls. Patients with HFpEF and NCD demonstrated reduced exercise capacity and impaired pulmonary function compared with controls, as evidenced by lower VO_2_, V_E_, and V_T_ and a higher V_E_ vs. VCO_2_ slope during peak exercise. Thus, these expired gas variables provided a moderate diagnostic ability to differentiate individuals with HFpEF from the controls. However, it is important to note that there is little question that the control population did not have symptoms of HF, and most would agree that this is not the cohort in which diagnostic evaluation is required. It is necessary to include a control group that also complains of dyspnea and in whom the disease is definitively ruled out to accurately evaluate diagnostic accuracy.

Despite substantial differences in the systolic and diastolic reserve capacity during exercise, we demonstrated that pulmonary function was similarly impaired in patients with HFpEF and NCD, except for peak VO_2_. This led to the poor diagnostic ability of the expired gas parameters in discriminating HFpEF from NCD (all AUCs < 0.61) and these data could be related to the study by Reddy et al.^19^. Pulmonary diseases are common comorbidities in patients with HFpEF, and diagnosis of HFpEF in this setting is often challenging. The inclusion of more patients with pulmonary diseases in the present study might have worsened the discriminative abilities, but the discriminative abilities were modest even after excluding NCD with significant pulmonary diseases. Further studies are needed to find alternative approaches to identify HFpEF in patients with dyspnea.

We found a limited diagnostic value of the expired gas parameters in distinguishing HFpEF from NCD. However, this does not deny the potential clinical value of CPET imaging for the evaluation and management of HFpEF. CPET provides an objective assessment of exercise capacity by measuring peak VO_2_. Since exercise stress echocardiography does not allow quantitative evaluation of exercise tolerance, its combination with expired gas anlaysis is of great merit in this regard. One of the goals of HF treatment is to improve functional capacity. Repeat measurements of expired gas data, especially peak VO_2_, allow assessment of responses to pharmacological interventions or lifestyle modifications in patients with HFpEF^26–30^. Moreover, expired gas analysis provides important prognostic information for patients with HFpEF. Peak VO_2_ and the V_E_ vs. VCO_2_ slope are associated with poor clinical outcomes in patients with HFpEF^31,32^.

The present study has several limitations. This was a single-center study conducted at a tertiary referral center, introducing a selection bias. All participants were referred for exercise stress testing, which also biased the results. The diagnosis of HFpEF was determined based on the HFA-PEFF algorithm, and a minority of the participants underwent exercise right heart catheterization, which is the current gold standard test. In the current study, expired gas data were obtained in the supine position using a stepped protocol because of simultaneous assessment with exercise stress echocardiography. This might have influenced the overall results, and our findings should be interpreted with caution. We used the septal e′ velocity to calculate E/e′ ratio, rather than the average value. However, septal E/e′ ratio is reported to be highly correlated with pulmonary capillary wedge pressure at rest and during exercise^5^.

## Conclusions

Expired gas analysis provided an simultaneous and objective assessment of exercise capacity during exercise stress echocardiography; however, its diagnostic value in identifying HFpEF among patients with dyspnea was limited. Beyond the application of exercise prescriptions and assessment of exercise capacity, further studies are warranted to determine the role of expired gas analysis in the evaluation and management of HFpEF.

## Supplementary Information

Supplementary Tables.

## Data Availability

The datasets generated during and/or analysed during the current study may be available from the corresponding author on reasonable request.
