# Supplementary material for: Diagnostic value of expired gas analysis in heart failure with preserved ejection fraction
Source: Sci Rep. 2023 Mar 16;13:4355. doi: 10.1038/s41598-023-31381-6 (PMC10020480; doi:10.1038/s41598-023-31381-6)
Supplement: Supplementary file 1 — Supplementary Tables. [file 41598_2023_31381_MOESM1_ESM.docx]

**SUPPLEMENTAL MATERIALS FOR:**

**Diagnostic Value of Expired Gas Analysis**

**in Heart Failure with Preserved Ejection Fraction**

Yuki Saito, MD, PhD^a,b*^, Masaru Obokata MD, PhD^a^, Tomonari Harada, MD, PhD^a*^

Kazuki Kagami, MD^a,c^, Makoto Murata, MD, PhD^d^, Hidemi Sorimachi, MD, PhD^a^ Toshimitsu Kato, MD, PhD^a^, Naoki Wada, MD, PhD^e^, Yasuo Okumura, MD, PhD^b^
Hideki Ishii, MD, PhD^a^

^a^Department of Cardiovascular Medicine, Gunma University Graduate School of Medicine, Maebashi, Gunma, Japan; ^b^Division of Cardiology, Department of Medicine, Nihon University School of Medicine, Tokyo, Japan; ^c^Division of Cardiovascular Medicine, National Defense Medical College, Tokorozawa, Saitama, Japan; ^d^Department of Cardiology, Gunma Prefectural Cardiovascular Center, Maebashi, Gunma, Japan; ^e^Department of Rehabilitation Medicine, Gunma University Graduate School of Medicine, Maebashi, Gunma, Japan

*These authors contributed equally to this work

**Running title:** Expired gas analysis in HFpEF

**Address for correspondence**:

Masaru Obokata, MD, PhD

Department of Cardiovascular Medicine

Gunma University Graduate School of Medicine,

3-39-22 Showa-machi, Maebashi, Gunma 371-8511, Japan

Tel.: +81-27-220-8145; Fax: +81-27-220-8158

E-mail address: [obokata.masaru@gunma-u.ac.jp](mailto:obokata.masaru@gunma-u.ac.jp)

**Supplemental Tabels**

**Supplemental Table 1: Baseline Characteristics** **in Patients with Sinus Rhythm**

|  | **Controls**  **(n=20)** | **NCD**  **(n=108)** | **HFpEF**  **(n=90)** | **P value** |
| --- | --- | --- | --- | --- |
| Age (years) | 68±7 | 65±13 | 74±7*^†^ | <0.0001 |
| Female, n (%) | 13 (65) | 68 (63) | 59 (65) | 0.92 |
| Body mass index (kg/m^2^) | 23.6±4.9 | 23.6±6.0 | 24.0±4.2 | 0.18 |
| ***Comorbidities*** |  |  |  |  |
| Coronary disease, n (%) | 1 (5) | 2 (2) | 13 (14)^†^ | 0.002 |
| Diabetes mellitus, n (%) | 1 (5) | 16 (14) | 27 (30)*^†^ | 0.004 |
| Hypertension, n (%) | 12 (60) | 62 (57) | 75 (83)*^†^ | 0.0002 |
| ***Medications*** |  |  |  |  |
| ACEI or ARB, n (%) | 8 (40) | 28 (26) | 38 (42)^†^ | 0.04 |
| Beta-blocker, n (%) | 5 (25) | 10 (9) | 23 (25)^†^ | 0.006 |
| Loop diuretics, n (%) | 1 (5) | 9 (8) | 25 (28)*^†^ | 0.0004 |
| ***Laboratories*** |  |  |  |  |
| BNP (pg/mL), n=123 | 32 (17, 55) | 31 (15, 54) | 81 (41, 183)*^†^ | <0.0001 |
| NT-pro BNP (pg/mL), n=64 | 131 (97, 188) | 128 (66, 170) | 294 (129, 519)^†^ | 0.001 |
| Red Blood Cell count, ×10^6^/μL | 4.24±0.44 | 4.35±0.50 | 4.07±0.59^†^ | 0.003 |
| Hemoglobin, g/dL | 12.9±1.5 | 13.1±1.4 | 12.2±1.7^†^ | 0.001 |
| Hematocrit, (%) | 39.6±4.3 | 40.4±4.1 | 37.7±5.1^†^ | 0.0007 |
| eGFR, mL/min/1.73 m^2^ | 66.3±21.3 | 68.1±17.7 | 57.1±23.9^†^ | 0.03 |
| ***Vital signs*** |  |  |  |  |
| Heart rate (bpm) | 71±15 | 75±13 | 71±11 | 0.32 |
| Systolic BP (mmHg) | 132±38 | 129±21 | 129±19 | 0.97 |
| Saturation (%) | 97±1 | 97±2 | 97±2 | 0.17 |
| ***LV structure and function*** | | |  |  |
| LV diastolic dimension (mm) | 43±5 | 42±6 | 43±5 | 0.47 |
| LV mass index (g/m^2^) | 76±12 | 75±19 | 92±22*^†^ | <0.0001 |
| LV ejection fraction (%) | 65±4 | 64±6 | 64±7 | 0.59 |
| LA volume index (mL/m^2^) | 30 (24, 31) | 23 (19, 28)* | 34 (26, 43)*^†^ | <0.0001 |
| E/e’ ratio (septal) | 10.1±2.7 | 9.4±2.6 | 14.5±5.1*^†^ | <0.0001 |
| PASP (mmHg) | 23±6 | 22±6 | 22±7 | 0.84 |
| RAP (mmHg) | 3±1 | 3±2 | 3±2 | 0.57 |
| ***Expired gas data*** |  |  |  |  |
| VO_2_ (mL/kg/min) | 4.2±1.1 | 4.1±1.3 | 3.9±0.9 | 0.13 |
| Respiratory rate (/min) | 14.6±5.3 | 16.8±5.5 | 16.5±6.3 | 0.17 |
| V_E_ (L/min) | 8.9±2.3 | 9.9±3.7 | 9.2±2.6 | 0.40 |
| V_T_ (mL) | 667±259 | 600±184 | 608±218 | 0.48 |

Data are mean ± SD, median (interquartile range), or n (%). Final column reflects overall group differences. *p<0.05 vs. Controls; ^†^p<0.05 vs. NCD. Abbreviations; ACEI,angiotensin-converting enzyme inhibitors; ARB, angiotensin-receptor blockers; BNP, B-type natriuretic peptide; BP, blood pressure; E/e’ ratio, the ratio of early diastolic mitral inflow to mitral annular tissue velocities; eGFR, estimated glomerular filtration rate; HFpEF, heart failure with preserved ejection fraction; LA, left atrial; LV, left ventricular; NCD, non-cardiac dyspnea; NT-proBNP, N-terminal pro B-type natriuretic peptide; PASP, pulmonary artery systolic pressure; and RAP, right atrial pressure; V_E,_ minute ventilation; VO_2_, oxygen consumption; V_T,_ tidal volume.

**Supplemental Table 2: Echocardiographic Measures and Expired Gas Data during Peak Exercise in Patients with Sinus Rhythm**

|  | **Controls**  **(n=20)** | **NCD**  **(n=108)** | **HFpEF**  **(n=90)** | **P value** |
| --- | --- | --- | --- | --- |
| Peak Watts (W) | 69±32 | 59±21 | 46±19*^†^ | <0.0001 |
| Exercise time (min) | 10.7±4.7 | 9.5±2.9 | 7.6±2.9*^†^ | <0.0001 |
| ***Vital signs*** |  |  |  |  |
| Heart rate (bpm) | 120±21 | 115±21 | 107±22^†^ | 0.01 |
| Systolic BP (mmHg) | 180±34 | 166±30 | 162±32 | 0.12 |
| Saturation (%) | 95±2 | 93±4 | 94±5 | 0.53 |
| ***Echocardiographic measures*** | |  |  |  |
| LV ejection fraction (%) | 71±5 | 71±6 | 70±7 | 0.71 |
| E-wave (cm/sec) | 122±20 | 109±23 | 126±29^†^ | 0.0001 |
| Septal mitral e’ (cm/sec) | 10.8±2.0 | 9.8±2.4 | 7.5±1.8*^†^ | <0.0001 |
| Septal mitral s’ (cm/sec) | 8.9±1.6 | 9.1±2.0 | 7.5±1.7*^†^ | <0.0001 |
| E/e’ ratio (septal) | 11.2±1.9 | 11.6±2.9 | 17.7±6.0*^†^ | <0.0001 |
| Cardiac output (L/min) | 7.5±1.5 | 7.1±2.0 | 6.7±2.1 | 0.06 |
| TV s’ (cm/sec) | 15.6±2.5 | 14.9±2.9 | 13.6±3.0^†^ | 0.005 |
| PASP (mmHg) | 43±12 | 42±11 | 43±13 | 0.75 |
| ***Expired gas data*** |  |  |  |  |
| VO_2_ (mL/min/kg) | 13.9±4.0 | 12.6±3.7 | 11.0±3.2*^†^ | 0.003 |
| O_2_ pulse (mL/min/beat) | 7.1±3.0 | 6.5±2.3 | 5.9±1.8 | 0.25 |
| RER | 1.20±0.16 | 1.18±0.15 | 1.12±0.13 | 0.13 |
| Respiratory rate (/min) | 30±6 | 32±7 | 33±8 | 0.21 |
| V_E_ (L/min) | 34.6±14.4 | 31.8±9.8 | 29.8±9.4 | 0.25 |
| V_T_ (mL) | 1170±475 | 1000±303 | 918±304 | 0.03 |
| Minimum V_E_/VCO_2_ (ratio) | 33.7±7.5 | 39.1±9.6 | 40.6±8.0*^†^ | 0.0005 |
| V_E_ vs. VCO_2_ slope | 31.9±8.6 | 36.5±9.3* | 39.6±8.6*^†^ | <0.0001 |

Data are mean ± SD. Final column reflects overall group differences. *p<0.05 vs. Controls; ^†^p<0.05 vs. NCD. TV, tricuspid valvular; RER, respiratory exchange ratio; VCO_2_, carbon dioxide volume; and other abbreviations as in Supplemental Table 1.

**Supplemental Table 3: Diagnostic Accuracy of Expired Gas Data in Patients with Sinus Rhythm**

|  | **Controls vs. HFpEF**  **AUC** | **P value** | **NCD vs. HFpEF**  **AUC** | **P value** | **NCD without ILD or COPD**  **vs. HFpEF**  **AUC** | **P value** |
| --- | --- | --- | --- | --- | --- | --- |
| *Peak Data* |  |  |  |  |  |  |
| Saturation (%) | 0.541 | 0.24 | 0.531 | 0.49 | 0.554 | 0.06 |
| VO_2_ (mL/min) | 0.688 | 0.001 | 0.627 | 0.0009 | 0.626 | 0.002 |
| VO_2_ (mL/min/kg) | 0.707 | 0.001 | 0.604 | 0.003 | 0.619 | 0.001 |
| %Predicted VO_2_ (%) | 0.613 | 0.07 | 0.516 | 0.91 | 0.535 | 0.31 |
| O_2_ pulse (mL/min/beat) | 0.585 | 0.03 | 0.559 | 0.04 | 0.560 | 0.07 |
| VCO_2_ (mL/min) | 0.701 | 0.0007 | 0.611 | 0.004 | 0.606 | 0.009 |
| Respiratory ratio (/min) | 0.605 | 0.08 | 0.552 | 0.23 | 0.578 | 0.06 |
| V_E_ (L/min) | 0.589 | 0.07 | 0.557 | 0.14 | 0.507 | 0.68 |
| V_T_ (mL) | 0.657 | 0.005 | 0.584 | 0.05 | 0.559 | 0.26 |
| V_E_ vs. VCO_2_ slope | 0.804 | <0.0001 | 0.610 | 0.02 | 0.671 | <0.0001 |
| Minimum V_E_/VCO_2_ (ratio) | 0.792 | 0.001 | 0.582 | 0.24 | 0.653 | 0.002 |
| V_D_/V_T_ (ratio) | 0.607 | 0.10 | 0.509 | 0.66 | 0.570 | 0.02 |

ILD, interstitial lung disease; COPD, chronic obstructive pulmonary disease; and other abbreviations as in Supplemental Tables 1 and 2.

**Supplemental Table 4: Baseline Characteristics** **(age-matched comparisons)**

|  | **Controls**  **(n=13)** | **NCD**  **(n=57)** | **HFpEF**  **(n=116)** | **P value** |
| --- | --- | --- | --- | --- |
| Age (years) | 73±2 | 75±4 | 74±7 | 0.34 |
| Female, n (%) | 6 (46) | 28 (49) | 67 (58) | 0.46 |
| Body mass index (kg/m^2^) | 24.9±4.8 | 22.7±3.9 | 24.8±6.4^†^ | 0.02 |
| ***Comorbidities*** |  |  |  |  |
| Coronary disease, n (%) | 1 (7) | 3 (5) | 16 (14) | 0.18 |
| Diabetes mellitus, n (%) | 2 (15) | 7 (12) | 35 (30)^†^ | 0.01 |
| Hypertension, n (%) | 10 (76) | 37 (64) | 96 (83)^†^ | 0.03 |
| Atrial fibrillation, n (%) | 10 (76) | 15 (26)* | 42 (36)* | 0.03 |
| ***Medications*** |  |  |  |  |
| ACEI or ARB, n (%) | 7 (53) | 20 (35) | 54 (47) | 0.23 |
| Beta-blocker, n (%) | 2 (12) | 7 (12) | 37 (32)^†^ | 0.008 |
| Loop diuretics, n (%) | 1 (7) | 6 (10) | 41 (36)*^†^ | 0.0002 |
| ***Laboratories*** |  |  |  |  |
| BNP (pg/mL), n=106 | 51 (28, 144) | 51 (25, 59) | 112 (46, 213)^†^ | 0.0005 |
| NT-pro BNP (pg/mL), n=74 | 159 (105, 383) | 128 (74, 186) | 511  (251, 1566)^†^ | <0.0001 |
| Red Blood Cell count, ×10^6^/μL | 4.49±0.58 | 4.31±0.55 | 40.9±0.61^†^ | 0.007 |
| Hemoglobin, g/dL | 13.6±1.9 | 13.1±1.5 | 12.3±1.8^†^ | 0.004 |
| Hematocrit, (%) | 41.6±5.7 | 40.2±4.5 | 37.9±5.5^†^ | 0.004 |
| eGFR, mL/min/1.73 m^2^ | 63.5±22.0 | 62.7±15.9 | 56.7±22.7 | 0.23 |
| ***Vital signs*** |  |  |  |  |
| Heart rate (bpm) | 68±15 | 72±12 | 73±13 | 0.22 |
| Systolic BP (mmHg) | 128±31 | 132±22 | 127±20 | 0.47 |
| Saturation (%) | 97±1 | 96±2 | 97±2 | 0.15 |
| ***LV structure and function*** | | |  |  |
| LV diastolic dimension (mm) | 44±4 | 42±6 | 44±6 | 0.14 |
| LV mass index (g/m^2^) | 82±16 | 76±17 | 93±23^†^ | <0.0001 |
| LV ejection fraction (%) | 64±2 | 63±6 | 63±7 | 0.85 |
| LA volume index (mL/m^2^) | 31 (24, 52) | 22 (20, 29)* | 36 (30, 47)*^†^ | <0.0001 |
| E/e’ ratio (septal) | 9.9±2.8 | 9.1±2.2 | 14.4±5.3*^†^ | <0.0001 |
| PASP (mmHg) | 24±4 | 22±6 | 24±10 | 0.19 |
| RAP (mmHg) | 4±2 | 3±1 | 4±3 | 0.01 |
| ***Expired gas data*** |  |  |  |  |
| VO_2_ (mL/kg/min) | 3.7±0.7 | 3.8±0.9 | 3.8±0.9 | 0.83 |
| Respiratory rate (/min) | 16.6±5.2 | 17.1±6.0 | 16.4±5.9 | 0.80 |
| V_E_ (L/min) | 9.2±2.1 | 9.7±3.5 | 9.5±2.5 | 0.92 |
| V_T_ (mL) | 587±163 | 582±179 | 613±205 | 0.86 |

Data are mean ± SD, median (interquartile range), or n (%). Final column reflects overall group differences. *p<0.05 vs. Controls; ^†^p<0.05 vs. NCD. Abbreviations as in Supplemental Table 1.

**Supplemental Table 5: Echocardiographic Measures and Expired Gas Data during Peak Exercise (age-matched comparisons)**

|  | **Controls**  **(n=13)** | **NCD**  **(n=57)** | **HFpEF**  **(n=116)** | **P value** |
| --- | --- | --- | --- | --- |
| Peak Watts (W) | 66±29 | 56±21 | 47±22^†^ | 0.004 |
| Exercise time (min) | 11.0±4.1 | 8.8±2.9 | 7.7±3.3*^†^ | 0.002 |
| ***Vital signs*** |  |  |  |  |
| Heart rate (bpm) | 117±22 | 109±22 | 109±23 | 0.57 |
| Systolic BP (mmHg) | 169±36 | 168±30 | 161±33 | 0.38 |
| Saturation (%) | 94±4 | 93±5 | 94±4 | 0.61 |
| ***Echocardiographic measures*** | |  |  |  |
| LV ejection fraction (%) | 73±5 | 71±7 | 69±8 | 0.09 |
| E-wave (cm/sec) | 120±17 | 105±21 | 127±30^†^ | <0.0001 |
| Septal mitral e’ (cm/sec) | 11.2±2.4 | 9.7±2.4 | 7.9±2.1*^†^ | <0.0001 |
| Septal mitral s’ (cm/sec) | 8.4±1.4 | 8.4±1.7 | 7.2±1.8^†^ | 0.0001 |
| E/e’ ratio (septal) | 11.1±2.5 | 11.5±3.2 | 17.1±6.1*^†^ | <0.0001 |
| Cardiac output (L/min) | 7.3±1.2 | 6.4±1.9 | 6.6±2.0 | 0.20 |
| TV s’ (cm/sec) | 13.8±2.1 | 14.3±2.6 | 12.7±3.3^†^ | 0.01 |
| PASP (mmHg) | 51±11 | 42±12* | 44±12 | 0.03 |
| ***Expired gas data*** |  |  |  |  |
| VO_2_ (mL/min/kg) | 13.9±3.6 | 12.2±3.8 | 10.9±3.4* | 0.009 |
| O_2_ pulse (mL/min/beat) | 7.5±2.9 | 6.6±2.5 | 6.0±2.0 | 0.14 |
| RER | 1.21±0.16 | 1.09±0.15 | 1.10±0.13 | 0.11 |
| Respiratory rate (/min) | 31±7 | 32±8 | 33±8 | 0.65 |
| V_E_ (L/min) | 36.7±13.8 | 31.3±10.0 | 29.9±9.8 | 0.14 |
| V_T_ (mL) | 1216±513 | 998±327 | 935±322 | 0.11 |
| Minimum V_E_/VCO_2_ (ratio) | 34.9±8.2 | 42.6±11.4* | 41.0±8.8* | 0.01 |
| V_E_ vs. VCO_2_ slope | 32.8±9.9 | 39.2±10.7* | 39.5±10.1* | 0.004 |

Data are mean ± SD. Final column reflects overall group differences. *p<0.05 vs. Controls; ^†^p<0.05 vs. NCD. Abbreviations as in Supplemental Tables 1 and 2.

**Supplemental Table 6: Diagnostic Accuracy of Expired Gas Data (age-matched comparisons)**

|  | **Controls vs. HFpEF**  **AUC** | **P value** | **NCD vs. HFpEF**  **AUC** | **P value** | **NCD without ILD or COPD**  **vs. HFpEF**  **AUC** | **P value** |
| --- | --- | --- | --- | --- | --- | --- |
| *Peak Data* |  |  |  |  |  |  |
| Saturation (%) | 0.517 | 0.93 | 0.546 | 0.32 | 0.519 | 0.39 |
| VO_2_ (mL/min) | 0.692 | 0.005 | 0.560 | 0.18 | 0.569 | 0.17 |
| VO_2_ (mL/min/kg) | 0.727 | 0.005 | 0.591 | 0.03 | 0.620 | 0.01 |
| %Predicted VO_2_ (%) | 0.702 | 0.01 | 0.563 | 0.12 | 0.598 | 0.05 |
| O_2_ pulse (mL/min/beat) | 0.657 | 0.02 | 0.548 | 0.11 | 0.539 | 0.23 |
| VCO_2_ (mL/min) | 0.712 | 0.001 | 0.547 | 0.25 | 0.557 | 0.22 |
| Respiratory ratio (/min) | 0.554 | 0.45 | 0.534 | 0.49 | 0.548 | 0.35 |
| V_E_ (L/min) | 0.648 | 0.03 | 0.551 | 0.36 | 0.524 | 0.67 |
| V_T_ (mL) | 0.660 | 0.009 | 0.556 | 0.23 | 0.536 | 0.45 |
| V_E_ vs. VCO_2_ slope | 0.775 | 0.006 | 0.514 | 0.85 | 0.571 | 0.13 |
| Minimum V_E_/VCO_2_ (ratio) | 0.758 | 0.007 | 0.518 | 0.35 | 0.547 | 0.41 |
| V_D_/V_T_ (ratio) | 0.651 | 0.07 | 0.502 | 0.94 | 0.547 | 0.25 |

Abbreviations as in Supplemental Tables 1, 2, and 3.

**Supplemental Table 7: Baseline Characteristics (excluding controls with** **elevated natriuretic peptide levels [BNP> 35 pg/mL or NT-proBNP >125 pg/m])**

|  | **Controls**  **(n=12)** | **NCD**  **(n=112)** | **HFpEF**  **(n=116)** | **P value** |
| --- | --- | --- | --- | --- |
| Age (years) | 67±6 | 65±13 | 74±7*^†^ | <0.0001 |
| Female, n (%) | 8 (66) | 69 (62) | 67 (58) | 0.74 |
| Body mass index (kg/m^2^) | 23.9±5.5 | 23.6±6.0 | 24.8±6.4 | 0.05 |
| ***Comorbidities*** |  |  |  |  |
| Coronary disease, n (%) | 0 (0) | 4 (3) | 16 (14)^†^ | 0.006 |
| Diabetes mellitus, n (%) | 0 (0) | 16 (14) | 35 (30)*^†^ | 0.0008 |
| Hypertension, n (%) | 9 (75) | 66 (59) | 96 (83)^†^ | 0.0003 |
| ***Medications*** |  |  |  |  |
| ACEI or ARB, n (%) | 5 (41) | 32 (29) | 54 (47)^†^ | 0.01 |
| Beta-blocker, n (%) | 3 (25) | 12 (11) | 37 (32)^†^ | 0.0004 |
| Loop diuretics, n (%) | 0 (0) | 10 (9) | 41 (36)*^†^ | <0.0001 |
| ***Laboratories*** |  |  |  |  |
| BNP (pg/mL), n=133 | 24 (12, 31) | 31 (16, 58) | 112 (46, 213)*^†^ | <0.0001 |
| NT-pro BNP (pg/mL), n=81 | 97  (97, 97) | 128  (68, 182) | 511  (251, 1566)^†^ | <0.0001 |
| Red Blood Cell count, ×10^6^/μL | 4.36±0.39 | 4.34±0.52 | 4.09±0.61^†^ | 0.004 |
| Hemoglobin, g/dL | 13.4±1.2 | 13.1±1.5 | 12.3±1.8^†^ | 0.002 |
| Hematocrit, (%) | 40.7±3.7 | 40.4±4.3 | 37.9±5.5^†^ | 0.001 |
| eGFR, mL/min/1.73 m^2^ | 62.7±14.5 | 67.7±17.7 | 56.7±22.7^†^ | 0.0009 |
| ***Vital signs*** |  |  |  |  |
| Heart rate (bpm) | 71±16 | 74±13 | 73±13 | 0.55 |
| Systolic BP (mmHg) | 138±45 | 129±21 | 127±20 | 0.88 |
| Saturation (%) | 98±1 | 97±2 | 97±2 | 0.07 |
| ***LV structure and function*** | | |  |  |
| LV diastolic dimension (mm) | 43±5 | 43±6 | 44±6 | 0.11 |
| LV mass index (g/m^2^) | 73±11 | 76±19 | 93±23*^†^ | <0.0001 |
| LV ejection fraction (%) | 66±3 | 63±6 | 63±7 | 0.40 |
| LA volume index (mL/m^2^) | 30 (25, 31) | 23 (19, 29)* | 36 (30, 47)*^†^ | <0.0001 |
| E/e’ ratio (septal) | 9.5±2.9 | 9.4±2.6 | 14.4±5.3*^†^ | <0.0001 |
| PASP (mmHg) | 21±6 | 22±6 | 24±10 | 0.26 |
| RAP (mmHg) | 3±1 | 4±2 | 4±3 | 0.13 |
| ***Expired gas data*** |  |  |  |  |
| VO_2_ (mL/kg/min) | 4.3±0.8 | 4.1±1.4 | 3.8±0.9 | 0.04 |
| Respiratory rate (/min) | 15.2±6.2 | 16.8±5.6 | 16.4±5.9 | 0.54 |
| V_E_ (L/min) | 9.2±2.3 | 9.9±3.7 | 9.3±2.5 | 0.73 |
| V_T_ (mL) | 696±329 | 601±181 | 613±205 | 0.72 |

Data are mean ± SD, median (interquartile range), or n (%). Final column reflects overall group differences. *p<0.05 vs. Controls; ^†^p<0.05 vs. NCD. Abbreviations as in Supplemental Table 1.

**Supplemental Table 8: Echocardiographic Measures and Expired Gas Data during Peak Exercise (excluding controls with** **elevated natriuretic peptide levels [BNP> 35 pg/mL or NT-proBNP >125 pg/m])**

|  | **Controls**  **(n=12)** | **NCD**  **(n=112)** | **HFpEF**  **(n=116)** | **P value** |
| --- | --- | --- | --- | --- |
| Peak Watts (W) | 82±34 | 59±21* | 47±22*^†^ | <0.0001 |
| Exercise time (min) | 12.0±4.8 | 9.4±3.0 | 7.7±3.3*^†^ | <0.0001 |
| ***Vital signs*** |  |  |  |  |
| Heart rate (bpm) | 125±19 | 115±21 | 110±24 | 0.01 |
| Systolic BP (mmHg) | 187±37 | 166±30 | 161±33* | 0.03 |
| Saturation (%) | 95±2 | 93±4 | 94±4 | 0.74 |
| ***Echocardiographic measures*** | |  |  |  |
| LV ejection fraction (%) | 72±4 | 71±6 | 69±8 | 0.15 |
| E-wave (cm/sec) | 128±22 | 110±24* | 127±30^†^ | <0.0001 |
| Septal mitral e’ (cm/sec) | 11.6±1.9 | 9.9±2.4 | 7.8±2.1*^†^ | <0.0001 |
| Septal mitral s’ (cm/sec) | 9.2±1.7 | 8.9±2.1 | 7.2±1.8*^†^ | <0.0001 |
| E/e’ ratio (septal) | 10.8±1.2 | 11.6±3.0 | 17.1±6.1*^†^ | <0.0001 |
| Cardiac output (L/min) | 7.8±1.1 | 7.0±2.0 | 6.6±2.0* | 0.02 |
| TV s’ (cm/sec) | 15.8±2.9 | 14.8±3.0 | 12.7±3.3*^†^ | <0.0001 |
| PASP (mmHg) | 42±10 | 42±11 | 44±12 | 0.30 |
| ***Expired gas data*** |  |  |  |  |
| VO_2_ (mL/min/kg) | 15.5±4.3 | 12.4±3.7* | 10.9±3.4*^†^ | 0.0001 |
| O_2_ pulse (mL/min/beat) | 7.8±3.3 | 6.5±2.3 | 6.0±2.0 | 0.17 |
| RER | 1.18±0.15 | 1.12±0.15 | 1.10±0.13 | 0.26 |
| Respiratory rate (/min) | 31±7 | 32±7 | 33±8 | 0.67 |
| V_E_ (L/min) | 38.2±16.6 | 31.8±9.9 | 29.9±9.8 | 0.08 |
| V_T_ (mL) | 1255±563 | 995±314 | 935±322 | 0.06 |
| Minimum V_E_/VCO_2_ (ratio) | 31.5±3.9 | 39.5±9.6 | 41.0±8.8*^†^ | 0.0004 |
| V_E_ vs. VCO_2_ slope | 30.0±3.4 | 37.0±9.5* | 39.5±10.1* | 0.0008 |

Data are mean ± SD. Final column reflects overall group differences. *p<0.05 vs. Controls; ^†^p<0.05 vs. NCD. Abbreviations as in Supplemental Tables 1 and 2.

**Supplemental Table 9: Diagnostic Accuracy of Expired Gas Data (excluding controls with** **elevated natriuretic peptide levels [BNP> 35 pg/mL or NT-proBNP >125 pg/m])**

|  | **Controls vs. HFpEF**  **AUC** | **P value** | **NCD vs. HFpEF**  **AUC** | **P value** | **NCD without ILD or COPD**  **vs. HFpEF**  **AUC** | **P value** |
| --- | --- | --- | --- | --- | --- | --- |
| *Peak Data* |  |  |  |  |  |  |
| Saturation (%) | 0.518 | 0.51 | 0.527 | 0.41 | 0.553 | 0.09 |
| VO_2_ (mL/min) | 0.745 | 0.0006 | 0.601 | 0.009 | 0.598 | 0.02 |
| VO_2_ (mL/min/kg) | 0.810 | 0.0002 | 0.609 | 0.002 | 0.623 | 0.002 |
| %Predicted VO_2_ (%) | 0.717 | 0.004 | 0.496 | 0.89 | 0.544 | 0.21 |
| O_2_ pulse (mL/min/beat) | 0.642 | 0.01 | 0.546 | 0.11 | 0.542 | 0.19 |
| VCO_2_ (mL/min) | 0.750 | 0.0004 | 0.596 | 0.01 | 0.590 | 0.02 |
| Respiratory ratio (/min) | 0.553 | 0.43 | 0.532 | 0.38 | 0.457 | 0.27 |
| V_E_ (L/min) | 0.659 | 0.01 | 0.555 | 0.15 | 0.511 | 0.72 |
| V_T_ (mL) | 0.664 | 0.006 | 0.566 | 0.18 | 0.539 | 0.52 |
| V_E_ vs. VCO_2_ slope | 0.826 | <0.0001 | 0.575 | 0.05 | 0.625 | 0.001 |
| Minimum V_E_/VCO_2_ (ratio) | 0.857 | <0.0001 | 0.567 | 0.24 | 0.628 | 0.005 |
| V_D_/V_T_ (ratio) | 0.684 | 0.02 | 0.536 | 0.32 | 0.594 | 0.009 |

Abbreviations as in Supplemental Tables 1, 2, and 3.
